# Senecavirus A Entry Into Host Cells Is Dependent on the Cholesterol-Mediated Endocytic Pathway

**DOI:** 10.3389/fvets.2022.840655

**Published:** 2022-04-08

**Authors:** Meiyu Jia, Mingxia Sun, Yan-Dong Tang, Yu-Yuan Zhang, Haiwei Wang, Xuehui Cai, Fandan Meng

**Affiliations:** ^1^State Key Laboratory of Veterinary Biotechnology, Harbin Veterinary Research Institute, Chinese Academy of Agricultural Sciences, Harbin, China; ^2^Heilongjiang Provincial Key Laboratory of Veterinary Immunology, Harbin Veterinary Research Institute, Chinese Academy of Agricultural Sciences, Harbin, China

**Keywords:** SVA, reporter virus, viral entry, pig ANTXR1, cholesterol

## Abstract

Senecavirus A (SVA), an important member of the *Picornaviridae* family, causes vesicular disease in pigs. Here, we generated an EGFP-expressing recombinant SVA re-SVA-EGFP, which exhibited similar growth kinetics to its parental virus. The reporter SVA was used to study the role of pig ANTXR1 (pANTXR1) in SVA infection in a porcine alveolar macrophage cell line (PAM-Tang cells). Knockdown of the pANTXR1 significantly reduced SVA infection and replication in PAM-Tang cells, while re-expression of the pANTXR1 promoted the cell susceptibility to SVA infection. The results indicated that pANTXR1 is a crucial receptor mediating SVA infection. Subsequently, the viral endocytosis pathways for SVA entry into pig cells were investigated and the results showed that cholesterol played an essential role in receptor-mediated SVA entry. Together, these results demonstrated that SVA entered into host cells through the pANTXR1-mediated cholesterol pathway. Our findings provide potential targets to develop antiviral drugs for the prevention of SVA infection in the pig population.

## Introduction

Senecavirus A (SVA), a single-stranded positive-sense RNA virus, belongs to the genus *Senecavirus* in the family *Picornaviridae* (1, 2). The SVA is a nonenveloped icosahedral virus. Though antibodies against SVA have been detected in mice, houseflies, cattle, buffalo (3–5), and pigs have been considered to be the only natural hosts of SVA (3). SVA has a single open reading frame (ORF) encoding a polyprotein that is processed to different structural and non-structural proteins, including leader protein (L^pro^), P1 (VP4, VP2, VP3, and VP1), P2 (2A, 2B, and 2C), and P3 (3A, 3B, 3Cpro, and 3D^pol^) (2). Structural proteins VP0, VP1, and VP3 initially form the pentamers of SVA particle, and then the precursor VP0 is further cleaved into VP2 and VP4 to assemble full capsids (6). SVA is an emerging swine vesicular disease, which shows similar symptoms to the diseases caused by foot-and-mouth disease virus (FMDV), swine vesicular disease virus (SVDV), or vesicular stomatitis virus (VSV), vesicular exanthema of swine virus (VESV) (7). The typical clinical symptoms of SVA are vesicular or ulcerative lesions on the snout, oral mucosa, coronary bands, and hooves. Other clinical signs include fever, lethargy, lameness, cutaneous hyperemia, and anorexia (8, 9). Since the first positive case of SVA infection in pigs was reported in Canada in 2007 (7), SVA outbreak incidents have been reported in the United States, Brazil, China, Colombia, Thailand, and other countries within a decade (10–14), indicating that SVA could be a potential threat to the global pig industry.

SVA is the first oncolytic picornavirus to be tested in humans and to penetrate solid tumors through the vascular system. Several studies have highlighted that SVA has excellent potential to be a safe and effective oncolytic virus in cancer therapy. The phase II clinical trial against small cell lung cancer is underway (15–19). At present, receptor-mediated SVA infection in human cells has been identified. It has been reported sialic acids are the key components that mediate SVA infection (20). Anthrax toxin receptor 1 (ANTXR1), also known as tumor endothelial marker 8 (TEM8), is a bacterial anthrax toxin that also enters the cell through this protein. It is also considered as an essential cellular receptor for SVA infection in human cells by genome-wide loss-of-function screens (21). Subsequently, the structure of the SVA-ANTXR1 complex through cryo-electron microscopy (cryo-EM) single-particle analysis and specific interaction sites between SVA and ANTXR1 is elucidated (22). A similar study shows that ANTXR1 mediates the SVA attachment and uncoating by the detailed structural analysis of SVA-ANTXR1 interactions (6). It is worth noting that pigs are the natural host of SVA infection. Many aspects of viral interactions with the host remain unknown. Therefore, the identification and function analysis of SVA receptors and the viral internalization pathway in porcine cells will help to understand the SVA pathogenesis and infection characteristics in the pig population.

Cholesterol is important for regulating membrane fluidity (23) and plays an essential role for maintaining the functions of lipid rafts that are dynamic microdomains in cellular membranes (24, 25). It has been shown that lipid microenvironments may interfere with both enveloped and non-enveloped virus entry by altering the clustering of receptors in cholesterol-rich subdomains (26–29). In addition to its involvment of virus entry and budding, cholesterol has been shown to be important in maintaining the stability and infectivity of the enveloped virus (30). Therefore, the lipid rafts and the cholesterol can be considered potential targets to inhibit virus infection (31, 32).

Here, we generated an infectious clone of a SVA expressing EGFP to analyze whether the pig ANTXR (pANTXR1) mediates SVA infection in porcine cells. The siRNA interference and knockout, as well as overexpression of ANTXR1, showed that ANTXR1 is the receptor for SVA infection. Moreover, a cholesterol-dependent endocytosis pathway for SVA internalization in porcine cells was analyzed. Our findings have shown that pANTXR1 is a receptor for SVA infection and indicated that downregulation of the amount of cholesterol in the host could be a potentially effective strategy for the prevention of SVA infection in the pig population.

## Materials and Methods

### Cells, Viruses, and Antibodies

The PAM-Tang cells, porcine alveolar macrophage cell line, had been identified and preserved in our laboratory (33). PAM-Tang cells were cultured in RPMI 1640 medium (Gibco, USA) supplemented with 50 μM β-mercaptoethanol, 1% L-glutamine (Gibco), 10% fetal bovine serum (FBS) (Clark) at 37°C under 5% CO_2_. Swine testicular (ST) cells, 293T cells, and hANTXR1 knockout Hela cells were maintained in Dulbecco's modified Eagle's medium (DMEM) (Gibco) supplemented with 10% FBS at 37°C in 5% CO_2_. The Senecavirus A strain (ZS) was identified and preserved in our laboratory. Virus stock was propagated by infection of ST cells at a low multiplicity of infection (MOI of 0.01) in DMEM. Supernatants were clarified by low-speed centrifugation (200 g, 10 min, room temperature) and stored at −80°C. Anti-β-actin and anti-TEM8 antibodies were purchased from Sigma. The SVA polyclonal antibodies against SVA were produced in rabbits. The SVA-specific mouse monoclonal antibody 2F5 (1:1,000) was kindly provided by Dr. Yongning Zhang, China Agricultural University.

### Plasmid Construction

**Figure 1:**
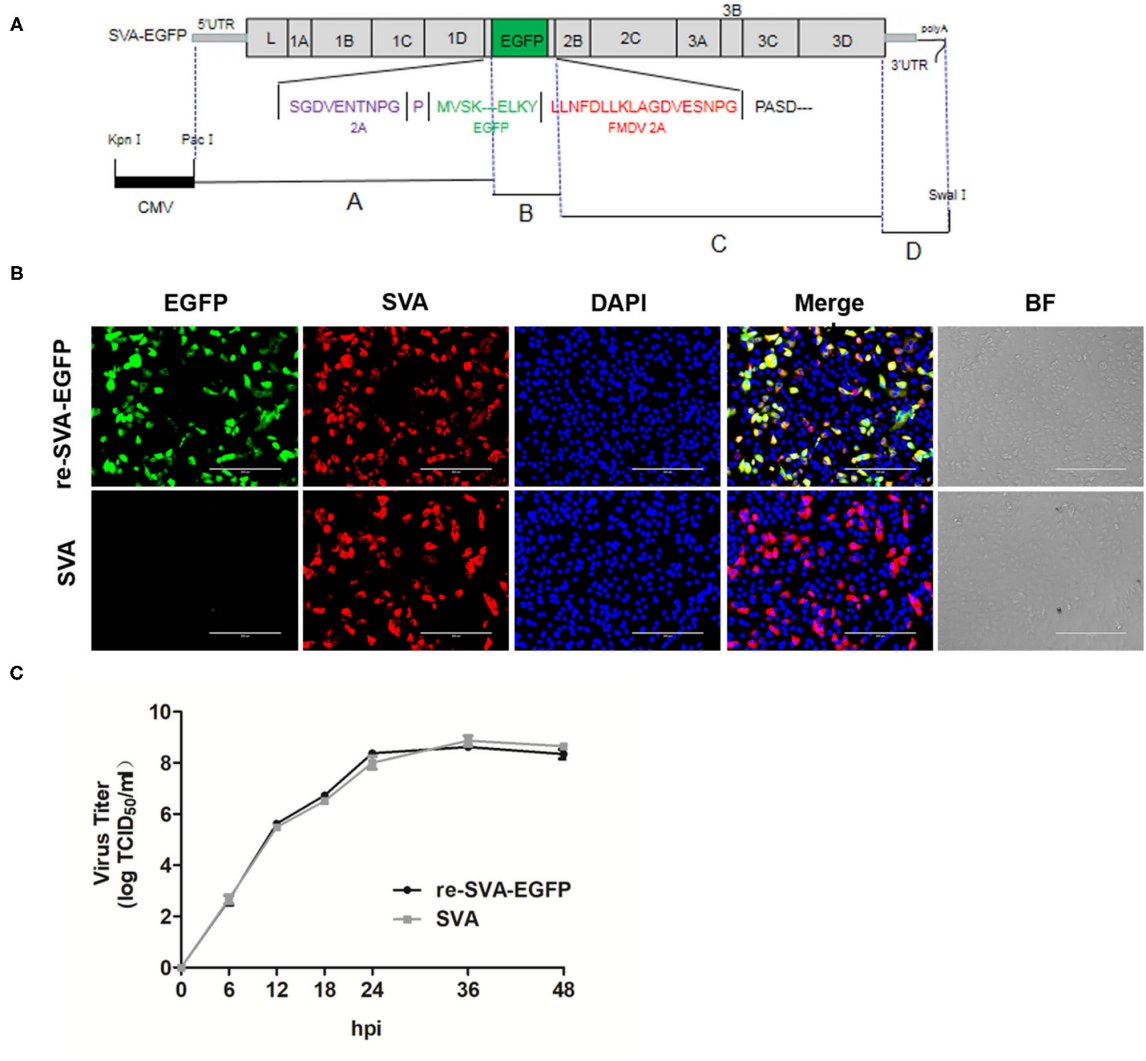
Schematic diagram of the full-length SVA genome and Generation of re-SVA-EGFP infectious clone. **(A)** The construction strategy of SVA clone. Four separate fragments, A to D, were assembled into the pBluescript II SK (+) vector using homologous recombination and the unique restriction enzyme sites. A hammerhead ribozyme and a HDV ribozyme was located at the 5 'and 3' ends of the SVA genome, respectively. The full-length genome is under the control of a CMV promoter. **(B)** Immunofluorescent assay for detecting SVA infection and EGFP expression. The SVA infected was labeled in red, EGFP was in green, and DAPI stained cell nucleus in blue. **(C)** One-step growth curve of re-SVA-EGFP infectious clone.

In order to construct a full-length SVA genomic cDNA clone, four separate fragments, named A to D, were amplified and assembled together. The full-length genomic cDNA clone was constructed into the plasmid vector pBluescript II SK (+), and the strategy was shown in Figure 1A. Cytomegalovirus (CMV) promoter was inserted upstream of the full-length SVA genomic cDNA, following the A to D separate fragments using the DNA recombination method. A hammerhead ribozyme sequence was inserted upstream of fragment A, while a hepatitis delta virus (HDV) ribozyme element was incorporated at the 3'-terminus of the viral genome. This full-length cDNA clone of SVA was designated as pSK-SVA-EGFP.

### Rescue the Recombinant Virus

The pSK-SVA-EGFP plasmid was transfected by DNA transfection reagent (Roche, Germany) following the manufacturer's protocol when 293T cells reached 90% confluency in a 6-well plate. At 48 h post-transfection, the supernatant from transfected 293T cells was collected and transferred to ST cells. Cytopathic effect (CPE) in ST cells was monitored daily after infection. When significant CPE was observed, the supernatants were harvested and clarified by low-speed centrifugation and stored at −80°C. The recombinant virus was named as re-SVA-EGFP.

### Virus Growth Curve and Titration

ST cells reached 100% confluency in a 6-well culture plate and were infected with parental SVA virus and re-SVA-EGFP virus at an MOI of 0.01 or mock-infected. After 2 h incubation, the unattached virus was removed and washed three times with Phosphate-Buffered Saline (PBS, pH 7.0), followed by the addition of fresh medium. The supernatant containing the virus was harvested at 6, 12, 18, 24, 36, and 48 hpi. For virus infectivity analysis, virus titers were determined by endpoint dilution titration on ST cells in 96-well plates. Briefly, 10-fold serial dilution steps were performed for each sample. Each dilution had 6 replicates and 100 μL/well was added onto confluent ST cells in a 96-well plate. Plates were incubated for an additional 48 h and the virus-induced CPE was analyzed. The Reed-Muench method determined the tissue culture infectious dose (TCID_50_) was 50%.

### RNA Extraction and Quantitative PCR

To evaluate viral load in cell and cell supernatant, SVA quantitative RT-PCR (qRT-PCR) was performed. Total RNA was extracted from cells and supernatant using the total RNA extraction kit and virus RNA kit (Qiagen, Germany), respectively. Subsequently, qPCR was performed using Premix Ex Taq (Probe qPCR) (TaKaRa, Japan). Quantitative PCR was performed under the following conditions: 95°C for 10 min for initial denaturation, followed by 40 cycles for 15 s at 95°C for denaturation, and for 1 min for annealing and collection of the PCR product.

### Immunofluorescence Assay

ST cells, PAM-Tang cells, and ANTXR1 knockout cells were plated in 24-well plate. When confluency reached 90%, cells were washed three times with PBS and infected with parental SVA virus and re-SVA-EGFP virus (MOI = 0.1). At the indicated time point, cells were fixed in 4% paraformaldehyde at room temperature for 20 min, incubated with 0.1 M glycine at room temperature for 5 min, washed three times with PBS, and then permeabilized with 0.2% Triton X-100 for 30 min. After washing with PBS three times, cells were blocked with 1% BSA at 37°C for 30 min, then incubated for 1 h at room temperature with primary antibodies. The primary antibodies used are as follow: SVA polyclonal antibodies were produced and preserved by our laboratory, antibodies against ANTXR1 (sigma, 1:200). Alexa Fluor 568-conjugated goat anti-mouse IgG (Thermo) was used as the secondary antibody; for nuclear visualization, cells were stained with DAPI (Sigma, USA). The images were acquired with an inverted fluorescence microscope (Zeiss Axio Observer 5, Germany).

### Western Blot Analysis

Treated and untreated PAM-Tang cells and ANTXR1 knockout Hela cells in a 6-well plate were infected with SVA (MOI = 0.1). After 24 hpi, cells were collected after being washed with PBS three times. The cell pellet was then incubated with RIPA Lysis Buffer (Solarbio, China) containing a protease inhibitor 0.1 mM phenylmethylsulfonyl fluoride for 30 min on ice. The supernatant was collected after centrifugation at 14,000 × g for 10 min at 4 °C, and the protein concentration was determined using the BCA assay (Thermo). Equal amounts of protein were separated on SDS-PAGE gels and then transferred to polyvinylidene fluoride (PVDF) membranes (ISEQ00010, Millipore, USA). The PVDF membranes were then blocked with 5% nonfat dry milk in PBS at room temperature for 1.5 h and incubated with primary antibodies at 4 °C overnight. The membrane was incubated with the secondary antibodies for 1 h after being washed three times with PBST (PBS: Tween- 20, 0.5‰ (V/V)). Immunoreactive bands were visualized using the enhanced chemiluminescence system (ECL, PerkinElmer Life Sciences).

### Small Interfering RNA Transfections

siRNA interference experiments were performed with PAM-Tang cells seeded in 6-well culture plates supplied with DMEM and 10% FBS 1 day prior to transfection with siRNAs. siRNAs targeted to porcine ANTXR1 (pANTXR1) were purchased from GenePharma company. The siRNA NC (sense siRNA 5′-UUCUCCGAACGUGUCACGUTT-3′ and antisense siRNA 5'-ACGUGACACGUUCGGAGAATT-3′) were used as the negative control. The siRNA1 (sense siRNA 5'-CCAGGAUCGCAGACAGUAATT-3′ and antisense siRNA 5'-UUACUGUCUGCGAUCCUGGTT-3′), siRNA2 (sense siRNA5'-GCUGAACCAUCCACCAUAUAUTT-3′ and antisense siRNA 5′-UUAUGGUGGAUGGUUCAGCTT-3′), and siRNA3 (sense siRNA5′-GCCGGAACAGGAGUAUGAATT-3′ and antisense siRNA 5'-UUCAUACUCCUGUUCCGGCTT-3′) were used to downregulate the pANTXR1. All siRNAs were delivered to PAM-Tang cells using X-tremeGENE siRNA Transfection Reagent (Roche) as the manufacturer's protocol. After 24 h interference with siRNAs, the cells were infected by the SVA virus (MOI = 0.1). The cells and supernatants were collected at 24 hpi for Western blot, viral titration, and qPCR analyses.

### pANTXR1 Overexpression Assays

Overexpression experiments were performed with PAM-Tang cells. Briefly, PAM-Tang cells were seeded in 6-well culture plates and supplied with DMEM and 10% FBS one day prior to transfection, pCAGGS-pANTXR1 plasmid or pCAGGS-empty vector was transfected using the same protocol as described above. At 24 h post-transfection, pANTXR1 re-expressing cells were inoculated with SVA at an MOI of 1, and the cells and supernatants were collected at 6 hpi for Western blot and viral titration.

### pANTXR1 Knockout by CRISPR/Cas9 Techniques

PAM-Tang cell lines were seeded in 6-well plates at a density of 6 × 10^5^ cells per well. When the cells grew to 90% confluency, pEGFP-N1 was mixed with pX330 with/without sgRNA sequences in the ratio of 1 to 1,000 and then introduced into the cells using a transfection reagent (Roche). The sgRNA sequences were sense sequence 5'CACCGAAGGGGGTCCAG CCTGCTA-3' and antisense sequence 5'-AAACTAGCAGGCTGGACCCCCTTC-3'. The sgRNA design website is https://cctop.cos.uni-heidelberg.de:8043. After 24 h post-transfection, the single-cell screened with EGFP was seeded into a 96-well plate with flow cytometry (BD Biosciences) and cultivated further. The knockout of the ANTXR1 gene was identified by DNA sequencing and real-time PCR. The SVA was added to ANTXR1 gene knockout cell lines, named KO PAM-Tang cells, at an MOI of 0.1, and cells and supernatants were collected for Western blot, viral titration, and RT-qPCR analysis.

### pANTXR1 Exogenous Re-Expression Assays

Re-expression experiments were performed with pANTXR1 knockout PAM-Tang cells and hANTXR1 knockout Hela cells, respectively, as previously described (21). Briefly, PAM-Tang KO cells and Hela KO cells were seeded in 6-well culture plates respectively, supplied with DMEM and 10% FBS 1 day prior to transfection, and pCAGGS-pANTXR1 plasmid was transfected using the same protocol as described above. After 24 h post-transfection, pANTXR1 re-expressing cells were inoculated with SVA at an MOI of 0.1, and then cells and supernatants were collected at 24hpi for Western blot, viral titration, and qPCR analyses.

### Cytotoxicity Test and Pharmacological Inhibitors

To test the cytotoxicity of Ethylisopropyl amiloride (EIPA) (MCE), methyl-β-cyclodextrin (MβCD) (MCZ), and chlorpromazine (CPZ) (SELLECK), the PAM-Tang cells were seeded in 96-well plates. When the confluency reached 100%, the cells were washed with PBS and treated with inhibitors at the indicated concentration for 45 min. Then 10 μL of CCK-8 solution was added to each well and incubated at 37°C for 1 h. An absorbance of 450 nm was measured. To test the effect of inhibitors on SVA internalization, PAM-Tang cells were pretreated with different concentrations of inhibitors for 45 min and then infected with SVA at MOI = 5 for 1.5 h. At 6 h post-infection, the cells and supernatants were collected for virus titration and Western blotting analysis, respectively. In addition, the inhibition effect of inhibitors was analyzed by immunofluorescence assay at 8 hpi.

### Statistical Analyses

All experiments were performed at least three times and results were expressed as the mean ± standard deviation (SD). Data was analyzed by *t*-test using GraphPad Prism version 9.00 (GraphPad, USA). A *P* value of <0.05 was considered significant.

## Results

### Generation of Re-SVA-EGFP

A cDNA encoding the full-length wild-type SVA genome was cloned into the pBluescript II SK (+) vector using the strategy shown in Figure 1. The recombinant plasmid pSK-SVA contains the 7,281 nucleotides full-length genome of SVA, flanked by a CMV promoter, a hammerhead ribozyme sequence, a poly (A) tail, and a hepatitis D virus (HDV) ribozyme element (Figure 1A). Meanwhile, to generate a recombinant virus expressing a reporter gene EGFP, a fusion protein of EGFP and FMDV 2A was inserted between 2A and 2B of the pSK-SVA backbone, resulting in pSK-SVA-EGFP. FMDV 2A NPG↓P allows self-cleavage of the peptide so that the EGFP-2A protein can release from SVA polyprotein. The plasmid pSK-SVA-EGFP was transfected into 293T cells to rescue recombinant SVA expressing EGFP. After 48 h post-transfection, the cell culture supernatant from the transfected cells was collected and passaged in ST cells. The rescued virus was designated as re-SVA-EGFP. The re-SVA-EGFP and its parental virus-infected ST cells were detected by immunofluorescence assay at 12 h post-infection using polyclonal antibodies against SVA virus particles. The results showed that most of the positive fluorescent cells had both EGFP and SVA virus proteins expression in re-SVA-EGFP infected cells. In contrast, no EGFP expression was observed from the wild-type SVA (SVA wt) (Figure 1B). Furthermore, the growth capacity between the re-SVA-EGFP virus and SVA wt was analyzed. The re-SVA-EGFP showed similar replication kinetics to SVA wt (Figure 1C), indicating re-SVA-EGFP has a good capacity for infection and replication when compared to that of the SVA wt.

### The pANTXR1 Interference Reduced SVA Replication in PAM-Tang Cells

**Figure 2:**
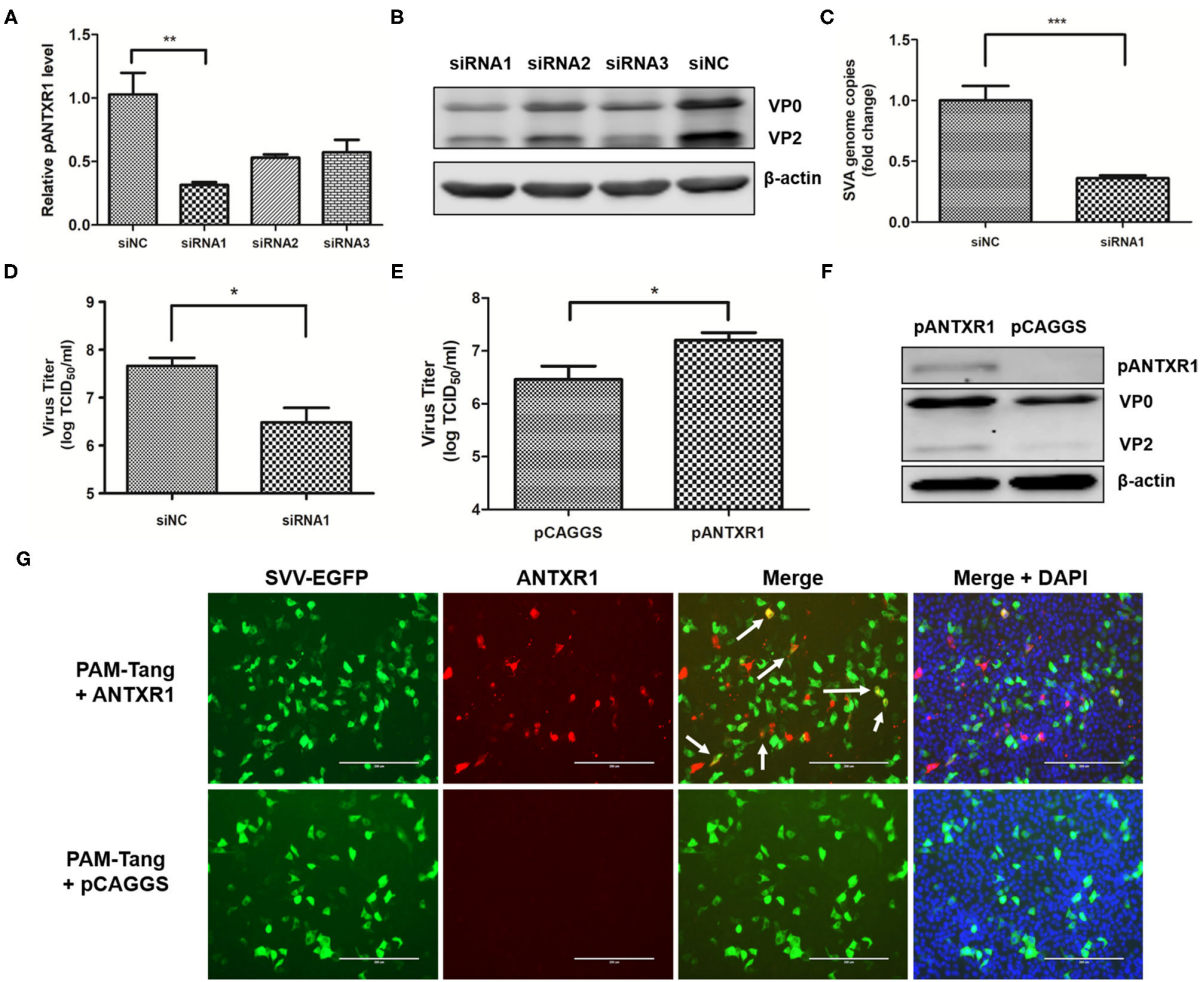
pANTXR1 interference reduced SVA replication in PAM-Tang cells. The SVA at an MOI of 0.1 was added to the PAM-Tang cells after siRNA interference. **(A)** The qPCR analysis of relative pANTXR1 mRNA levels changes in PAM-Tang cells (**p < 0.01). **(B)** The Western blot assay for the expression of SVA VP2 and VP0 proteins. **(C)** The qPCR analysis of SVA copy numbers in the supernatant (***p < 0.001). **(D)** The TCID_50_ assay for SVA titers in supernatants post siRNA1 interference (*p < 0.05). **(E)** The TCID_50_ assay for SVA titers in pANTXR1 overexpressed PAM-Tang cells at 6 hpi (*p < 0.05). **(F)** The western blot assay for the expression of SVA VP2, VP0 proteins and pANTXR1 from pANTXR1 overexpressed PAM-Tang cells at 6 hpi. **(G)** Immunofluorescent assay for pANTXR1 (in red) and EGFP (in green) expression level in the pANTXR1 overexpressed PAM-Tang cells, and the colocalization of pANTXR1 with EGFP was indicated by arrow. Bars, 200 μm.

To determine whether pANTXR1 was involved in SVA infection, the expression level of the pANTXR1 was downregulated by small interfering RNA (siRNA) interference in PAM-Tang cells. At 24 h of siRNA interference, the cells were collected for interference efficiency by the RT-qPCR detection. The results showed the siRNA1 significantly downregulated 70% of ANTXR1 level compared with the siRNA negative control (siNC) (*P* < 0.01) (Figure 2A). Meanwhile, the cell lysates were collected for Western blot analysis using anti-VP2 monoclonal antibody. The results showed that VP2 and VP0 protein expression was decreased when cells were transfected with siRNAs (Figure 2B). Furthermore, after 24 h of siRNA interference, the SVA (MOI = 0.1) was added to the siRNA1 interfered cells and the SVA genome copies and virus titer in the supernatant were determined. Compared with the siNC, the SVA copy number in the supernatant of the siRNA1 group significantly reduced (*p* < 0.001) (Figure 2C), and the infectious SVA titer in the supernatant also decreased (Figure 2D). These results indicated that pANTXR1 interference inhibits SVA replication in PAM-Tang cells.

### Overexpression of the pANTXR1 Increased SVA Infectivity

Overexpression of viral receptors on the cell surface may promote virus infection. Here, the effect of pANTXR1 overexpression on SVA proliferation in PAM-Tang cells was analyzed. At 24 h post-transfection, the cells were infected with SVA-EGFP virus for 6 hours, then the cells and supernatants were collected for further analysis. As shown in Figure 2E, the infectious SVA viral particles in the pANTXR1 overexpressed PAM-Tang cells were significantly higher than that of the empty vector (P<0.05). The overexpression of pANTXR1 was detected by WB (Figure 2F), which was associated with a slight increase in the amount of SVA VP0 and VP2 proteins. Besides, the amount of viral VP0 protein was much higher than VP2 protein at 6hpi. The overexpressed pANTXR1, but not the endogenously expressed pANTXR1, was detected by specific antiboby by immunofluorescence assay, which was consistent with WB detection results. The pANTXR1 protein labled in red was detected only in pCAGGS-pANTXR1 plasmid transfected cells, and the colocalization of pANTXR1 and EGFP was observed indicated by the arrow in Figure 2G. These results showed that pANTXR1 is able to mediate SVA infection.

### The pANTXR1 Knockout Suppressed SVA Replication in PAM-Tang Cells

**Figure 3:**
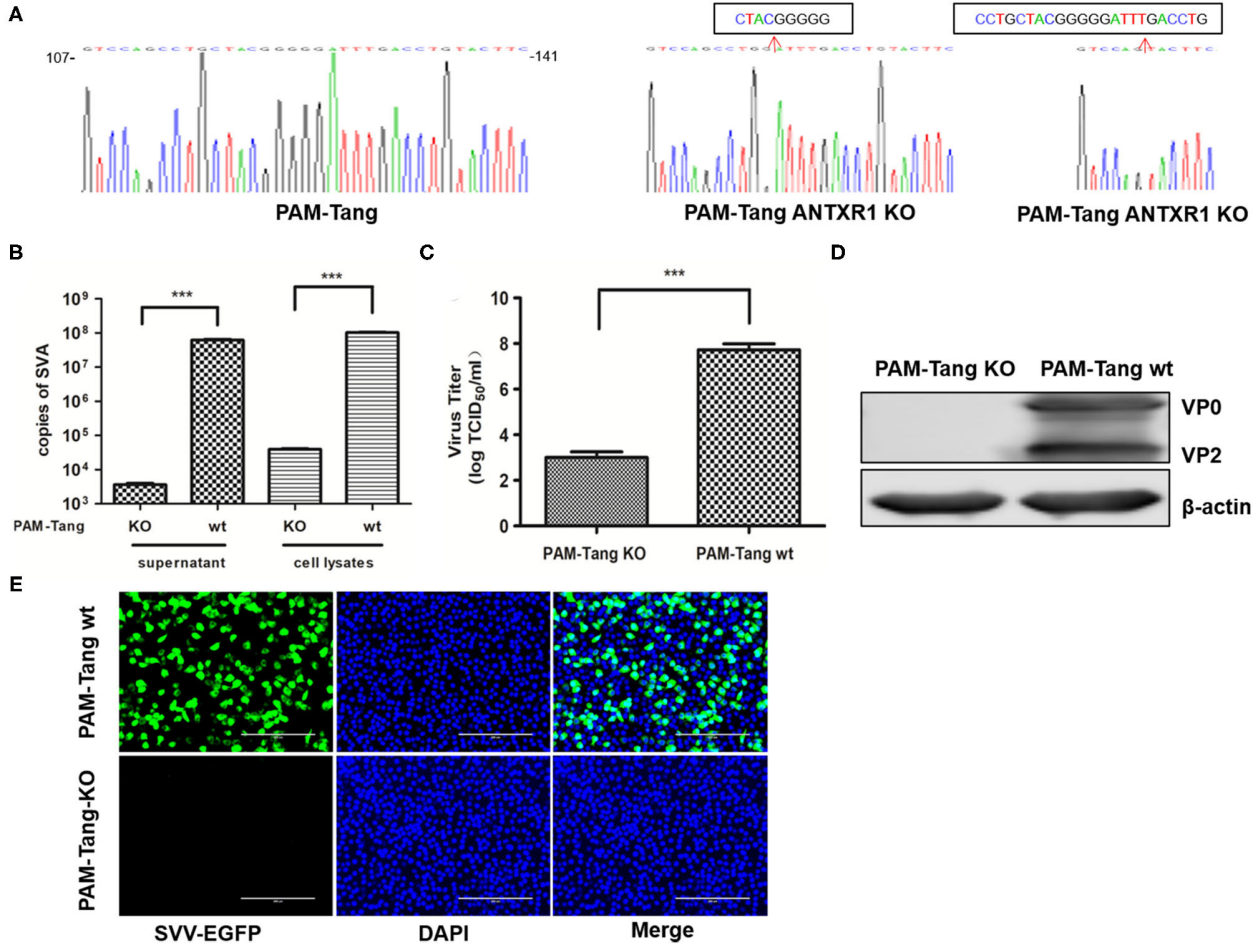
pANTXR1 knockdown suppressed SVA replication in PAM-Tang cells. **(A)** The genomic sequences around the target sites of WT and PAM-Tang ANTXR1 KO cells. **(B)** The qPCR analysis of SVA copy numbers in supernatant and cell lysates (***p < 0.001). **(C)** The TCID_50_ assay for SVA titers (***p < 0.001). **(D)** The Western blot assay for the expression of SVA VP2 and VP0 proteins. **(E)** The EGFP (in green) expression level in the PAM-Tang ANTXR1 KO cells and wild type cells. Bars, 200 μm.

To further confirm the above observation, a pANTXR1 knockout PAM-Tang cell line was established using CRISPR/Cas9 techniques. The pANTXR1 knockout PAM-Tang cell was named PAM-Tang KO. The sequencing results showed that there was a deletion of 8 and 23 nucleotides in the target region of PAM-Tang KO cells (Figure 3A). The SYBR Green I real-time RT-qPCR method was performed to detect whether the pANTXR1 was completely knocked out, the β-actin as an internal standard, using specific primers that the upstream primer pANTXR1KO-F included eight nucleotides knocked out (Supplementary Table 1). The PAM-Tang WT cells melting-curve showed a unique absorption peak, while the PAM-Tang KO cells had none (Supplementary Figure 1). The PAM-Tang KO cells were infected with SVA at an MOI of 0.1, and the wild-type PAM-Tang groups (PAM-Tang wt) were used as controls. The results showed that the absence of pANTXR1 notably suppressed SVA replication and the SVA copy number significantly dropped off at 24 hpi (*p* < 0.001) (Figure 3B) and the infectious virus titer in the supernatant was reduced by more than 10, 000 fold in PAM-Tang KO cells at 24 hpi (*P* < 0.001) (Figure 3C). Meanwhile, no expression of the viral structural protein (VP2) was detected by WB (Figure 3D), indicating that no SVA infection occurred in PAM-Tang KO cells. Furthermore, PAM-Tang KO and PAM-Tang wt were infected by re-SVA-EGFP at an MOI of 0.1, and the expression of EGFP was monitored by IFA at 12hpi (Figure 3E). The results showed that no EGFP expression was detected in the PAM-Tang KO cell line. These results demonstrated that knockdown of the pANTXR1 significantly reduce SVA replication in PAM-Tang cells.

### Exogenous Re-Expression of the PANTXR1 Recovered SVA Infectivity in PAM-Tang KO Cells

**Figure 4:**
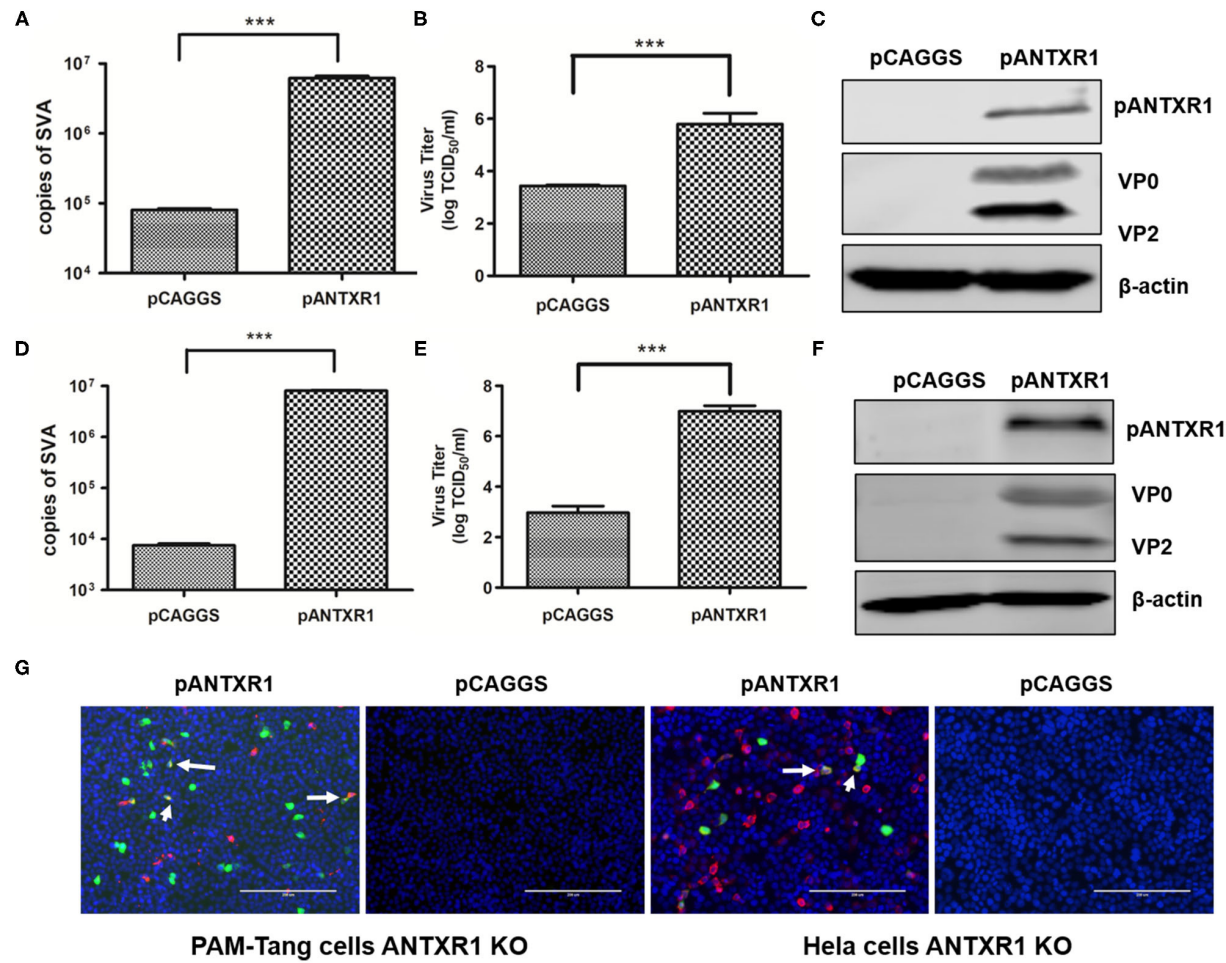
Exogenous re-expression of the pANTXR1 promoted SVA infectivity. pCAGGS-pANTXR1 plasmid was transfected into Hela KO cells and PAM-Tang KO cells, and the pANTXR1 re-expressing cells were inoculated with SVA at an MOI of 0.1 **(A)** The qPCR analysis of SVA copy numbers in the Hela KO cells (***p < 0.001). **(B)** The TCID_50_ assay for SVA titers in Hela KO cells (***p < 0.001). **(C)** The western blot assay for the expression of SVA VP2 and VP0 proteins and pANTXR1 from Hela KO cells. **(D)** The qPCR analysis of SVA copy numbers in the PAM-Tang KO cells (***p < 0.001). **(E)** The TCID_50_ assay for SVA titers in PAM-Tang KO cells (***p < 0.001). **(F)** The western blot assay for the expression of SVA VP2 and VP0 proteins and pANTXR1 from PAM-Tang KO cells. **(G)** Immunofluorescent assay for pANTXR1 (in red) and EGFP (in green) expression level in the pANTXR1 re-expressed PAM-Tang KO cells and Hela KO cells. The colocalization of pANTXR1 with EGFP was indicated by arrow. Bars, 200 μm.

To further analyze whether pANTXR1 can enhance cell susceptibility to SVA infection, full-length pig ANTXR1 protein was exogenous re-expressed in PAM-tang KO cells to recover the wild type cell phenotype and a human ANTXR1 protein knockout Hela cell line (Hela KO) was used as a control cell to avoid the effect of other porcine proteins. PAM-Tang KO cells and Hela KO cells were transfected with pCAGGS-pANTXR1, and the empty vector was used as the negative control. SVA (MOI = 0.1) was added to the cells at 24 h post-transfection. The SVA genomes, infectious virus titer, and viral protein expression level were determined at 24 hpi. The results showed that SVA genomic RNA was significantly increased in both pANTXR1 overexpressed PAM-Tang KO cells and Hela KO cells (*p* < 0.001) (Figures 4A,D). In addition, the virus titer in the supernatant enhanced ~1,000 fold in pANTXR1 overexpressed PAM-Tang KO cells (*P* < 0.001) (Figure 4B), and around 100 fold in that of Hela KO cells (*p* < 0.001) (Figure 4E). Furthermore, the expression of pANTXR1, viral structural protein VP0, and VP2 were able to be detected in pANTXR1 re-expressed PAM-Tang KO cells and Hela KO cells, respectively (Figures 4C,F). The pANTXR1 re-expressed PAM-Tang KO cells and Hela KO cells were infected by re-SVA-EGFP at an MOI of 0.1 and analyzed by IFA at 12 hpi. The results showed that the expression of EGFP was detected in either of pANTXR1 re-expressed cell lines instead of the empty vector transfected cells and colocalization of pANTXR1 labeled in red with EGFP was observed, which were indicated by arrow (Figure 4G). These results suggested that exogenous re-expression of pANTXR1 in ANTXR1 knockout cells can recover the cell susceptibility to SVA infection, indicating that pANTXR1 is essential for SAV infection in PAM-Tang cells.

### SVA Internalization Depends on Membrane Cholesterol

**Figure 5:**
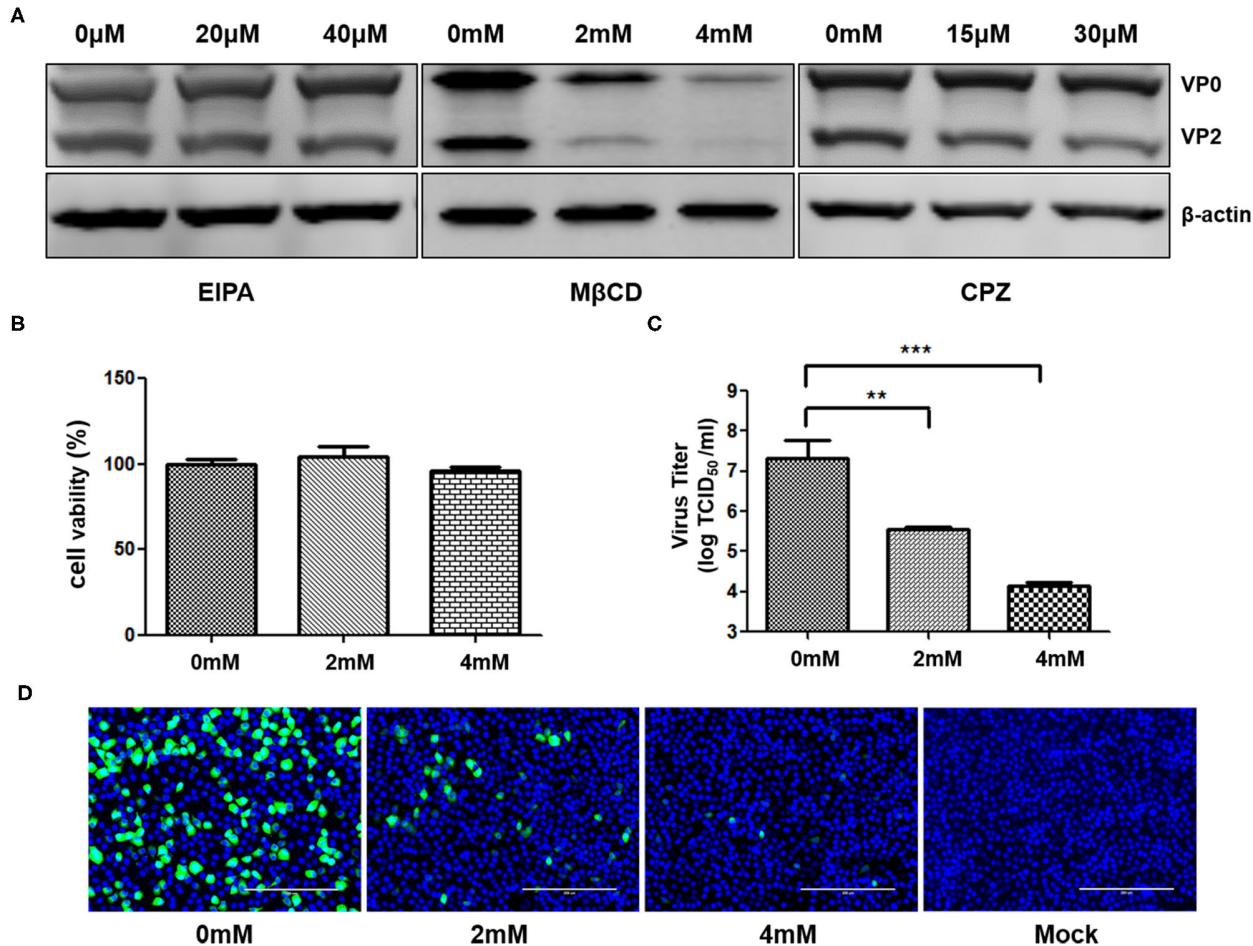
SVA internalization depends on membrane cholesterol. PAM-Tang cells were pretreated with inhibitors prior to SVA infection. **(A)** The Western blot analysis for the expression of SVA VP2 and VP0 proteins from PAM-Tang cells that were pretreated with inhibitors. **(B)** The cell viability test assay. **(C)** The TCID_50_ assay for viral titers (**P < 0.01, ***P < 0.001). **(D)** Immunofluorescent assay for EGFP (in green) expression level in the cells pretreated with MβCD. Bars, 200 μm.

To investigate the entry of SVA into porcine cells, different internalization pathways were analyzed. The PAM-Tang cells were treated with EIPA, MβCD, or CPZ prior to SVA infection (MOI = 5). Subsequently, the cell lysates were collected at 6 hpi to detect the viral protein expression. The SVA VP2 and VP0 protein decreased in MβCD (4 mM) pretreated cells, while the EIPA and CPZ pretreatment showed no effect on the amount of viral protein expression (Figure 5A). MβCD, with indicated concentration, showed no cytotoxic effect on PAM-Tang cells and the cell viability analysis was shown in Figure 5B. Besides, the infectious SVA titer in the supernatant was detected and the viral titer in MβCD pretreated cells was significantly reduced (*P* < 0.01) (Figure 5C). When the cells were treated with 4 mM MβCD, the viral titer decreased by more than 1,000 fold. Furthermore, the PAM-Tang cells were pretreated with or without MβCD at indicated concentrations and infected by re-SVA-EGFP at an MOI of 1, and the expression of EGFP was monitored at 8hpi by confocal microscope. The EGFP expression level in MβCD pretreated group was much lower than in the no MβCD pretreatment group (Figure 5D). Besides, only a few EGFP signals were barely detected in 4 mM MβCD treating group (Figure 5D). These results indicated that MβCD significantly inhibits SVA internalization in PAM-Tang cells. Together, cholesterol plays an important role in SVA entry into PAM-Tang cells.

## Discussion

Replication-competent reporter-expressing system of some viruses had been applied to the study of virus entry, including SARS-CoV-2, Marek's disease virus, and measles virus (34–36). In this study, a recombinant SVA expressing EGFP was successfully rescued by reverse genetics technology, which showed similar replication characteristics when compared with its parental virus. It has been shown that polymerase fidelity contributes to the genetic stability of the SVA expressing GFP gene (37). The reporter expressed that recombinant virus is a good tool to detect viral infection without being interfered by secondary labeling directly. Therefore, re-SVA-EGFP was used to detect the SVA entry in porcine cells.

Anthrax toxin receptor is highly expressed in many human cancer cells, which shows common characteristics with many related proteins-immunoglobulin superfamily that mediates picornaviruses infection. It has been identified as a receptor of SVA infection on human neuroendocrine cancer cells (21). Therefore, SVA is considered as oncolytic viruses and shows potential in cancer therapy. However, SVA is one of the important pathogens that cause vesicular disease in the pig population, and the interactions between SVA and host cells should be of more concern. Since the pANTXR1 share high homology with hANTXR1 (around 97%), we are interested in investigating whether pANTXR1 is able to mediate SVA infection in porcine cells. It has been confirmed that there are 12 specific interaction sites between SVA and hANTXR1 (22). Among them, R88Q and D156E mutations occur in the pANTXR1, and residue R has different physicochemical properties from Q. In this study, we used the PAM-Tang cells to investigate whether the pANTXR1 is the SVA receptor in pigs. We found that reducing the amount of pANTXR1 expression on PAM-Tang cells by RNA interference interfered with SVA infection. Subsequently, the PAM-Tang pANTXR1 knockout cell line (PAM-Tang KO cells) showed very low susceptibility for SVA infection, indicating that pANTXR1 is essential for SVA infection. Since it has been reported that removal or blocking of sialic acid can moderately reduce the SVA infection in glioma cells (20), sialic acid could act as an alternative mediator for SVA infection in pANTXR1 knock out cells. Furthermore, the pANTXR1 protein overexpression in PAM-Tang cells or re-expression in PAM-Tang KO cells increased the susceptibility of PAM-Tang cells to SVA infection.

Our results showed that the colocalization of overexpression of pANTXR1 protein and SVA-infected cells is limited. However, the viral titer was significantly increased in pANTXR1 overexpressed cells and the exogenous re-expressed pANTXR1 KO cells. In addition, the location of overexpressed pANTXR1 protein was changed in SVA infected cells, not expressing on the cell surface but translocating to the cytoplasm. We speculated that the cellular membrane protein must undergo proper folding and glycosylation modification post sorting on the cell membrane to mediate viral infection (38–40). Some of the pANTXR1 overexpressed on cells may be misfolded or have incorrect glycosylation to mediate SVA attachment properly. Conversely, it has been confirmed that ANTXR1 mediates SVA attachment and the uncoating process during viral infection, and the SVA-ANTXR1 complex undergoes a series of reconfiguration of the pentameric capsid assemblies, which leads to the release viral genome under acidic conditions in late endosome (6). The antibodies against ANTXR1 may not recognize the SVA-ANTXR1 complex protein post reconfiguration and the antigenic site may be masked during viral genome release process. Therefore, only partial pANTXR1 expression can be detected in SVA-infected cells. In addition, the misfolded pANTXR1 may be unable to complete the uncoating process either.

Furthermore, we confirmed that SVA did not infect the hANTXR1 protein knockout Hela cell line (Hela KO), which was used as a control to avoid the effect of other porcine proteins on SVA infection. We observed pANTXR1 also mediated SVA infection in Hela KO cells, which indicated that the interaction sites Q88 and E156 in pANTXR1 showed no effect on mediating SVA infection in both human and porcine cells. These results confirmed that pANTXR1, as an important cellular receptor, plays a crucial function for SVA infection in PAM-Tang cells. Viral receptors' expression on host cells not only determine the viral tropism to the host species or tissue, but also provide host targets for antiviral strategies. So far, multiple cellular viral receptors have been aimed as antiviral targets (41, 42). For example, mimicking the influenza receptor, sialic acid has demonstrated the inhibition of the influenza virus infection in a clinical setting. Therefore, pANTXR1 could be a potentially effective target for the prevention of SVA infection in the pig population.

Different viruses enter cells via different cellular pathways. Macropinocytosis, lipid rafts, and the classical clathrin-mediated endocytosis are confirmed to be able to mediate virus entry. Thus far, the endocytosis pathways for SVA internalize the porcine cells remains unclear. Three inhibitors selectively blocking different endocytosis were used to analyze the SVA entry pathway. We found that the methyl-β-cyclodextrin (MβCD), a pharmacological inhibitor of cholesterol-mediated endocytosis (43), significantly reduced the SVA infection in a dose-dependent manner in PAM-Tang cells. It has been investigated that some picornavirus takes advantage of macropinocytosis to internalize cells (44). We found that the selective macropinocytosis blocker ethylisopropyl amiloride (EIPA) showed no inhibition effect on SVA infection in PAM-Tang cells. Neither the CPZ inhibited the SVA entry pathway via the classical clathrin-mediated endocytosis. These results indicated that cholesterol-mediated endocytosis, instead of the macropinocytosis or clathrin-mediated endocytosis, is involved in the internalization of SVA into porcine host cells. During the viral initiated infection stage, the interactions between cellular components and viral particles play a crucial role (45). Cholesterol may directly affect SVA particle interactions with the cellular membrane. Since the amount of cholesterol is important for maintaining cellular membrane fluidity, depletion of cholesterol may also alter SVA particle diffusion within the lipid bilayer. Furthermore, treatment of MβCD may disrupt lipid rafts that are membrane microdomains enriched in cholesterol, sphingolipids, and glycolipids, and play a significant role in the process of extracellular signal transmembrane transmission (43, 46). Therefore, disruption of lipid rafts may interfere with the extracellular signal transmembrane transmission induced by SVA infection. It has been reported that lipid rafts and cholesterol take part in the virus entry process. Except for enveloped viruses, some non-enveloped viruses have been shown to enter host cells in a cholesterol-dependent manner, including poliovirus, Coxsackievirus, and noroviruses (47–49). Lipid rafts are also involved in the viral release process (50), and further investigation is needed as to whether cholesterol depletion affects SVA budding needs.

In conclusion, the identification of cellular receptors for SVA infection in the natural host is important to understand the interactions between pathogens and host and for developing potential novel antiviral strategies. In this study, we identify that pANTXR1 is a crucial receptor for SVA infection in pig cells, and knocking down pANTXR1 significantly decrease SVA infection and replication. In addition, we show that cholesterol is involved in the internalization of SVA, and the viral entry is dependent on the cholesterol-mediated endocytic pathway. Our findings provide two potentially effective targets for the prevention of SVA infection in the pig population.

## Data Availability Statement

The original contributions presented in the study are included in the article/Supplementary material, further inquiries can be directed to the corresponding authors.

## Author Contributions

MJ and MS performed the experiments. FM and XC conceived the study and designed the experimental procedures. Y-YZ contributed reagents and materials. MJ and Y-DT analyzed the data. MJ, MS, and HW wrote the first draft of the manuscript. FM, Y-DT, and XC wrote sections of the manuscript. All authors contributed to manuscript revision, read, and approved the submitted version.

## Funding

This work was supported by grant from the National Natural Science Foundation of China to FM (32002249) and the Natural Science Foundation of Heilongjiang Province to MS (QC2018030).

## Conflict of Interest

The authors declare that the research was conducted in the absence of any commercial or financial relationships that could be construed as a potential conflict of interest. The reviewer TS declared a shared affiliation with the authors to the handling editor at the time of the review.

## Publisher's Note

All claims expressed in this article are solely those of the authors and do not necessarily represent those of their affiliated organizations, or those of the publisher, the editors and the reviewers. Any product that may be evaluated in this article, or claim that may be made by its manufacturer, is not guaranteed or endorsed by the publisher.
