# Supplementary material for: Senecavirus A Entry Into Host Cells Is Dependent on the Cholesterol-Mediated Endocytic Pathway
Source: Front Vet Sci. 2022 Apr 8;9:840655. doi: 10.3389/fvets.2022.840655 (PMC9040607; doi:10.3389/fvets.2022.840655)
Supplement: Supplementary file 1 [file Table_1.DOCX]

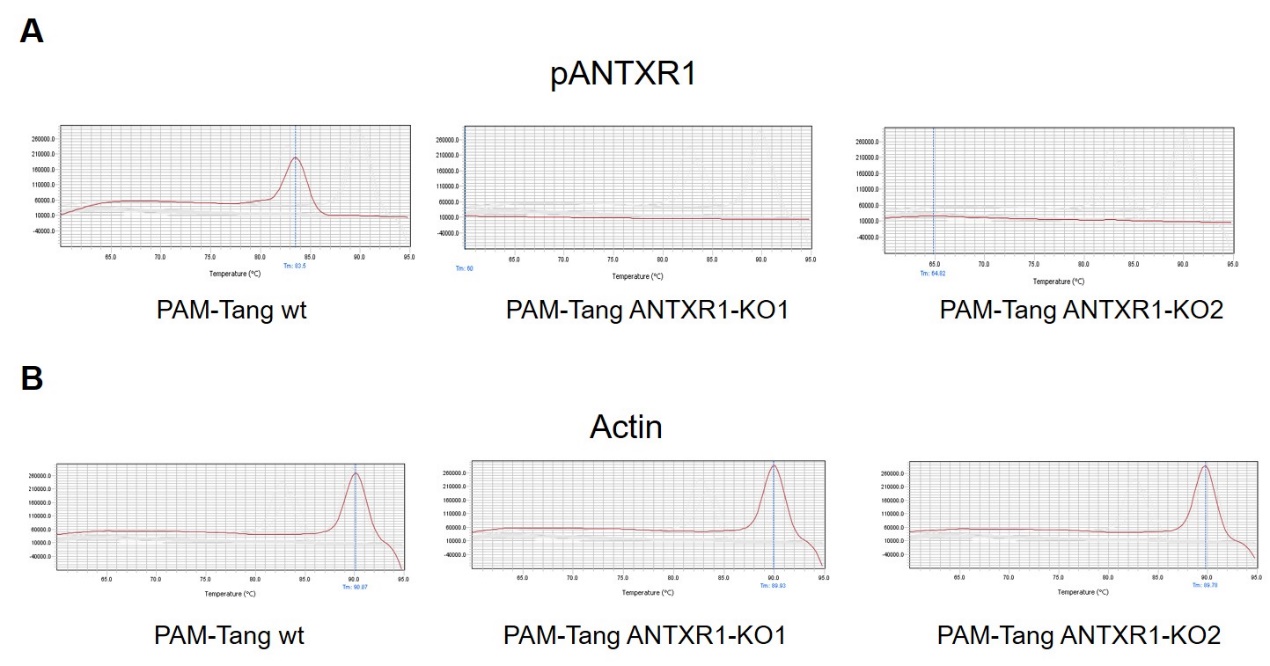


**Figure S1. The pANTXR1 was completely knocked out by the detection of the SYBR Green I real-time RT-qPCR method.** (A) The melting-curve of PAM-Tang wt cells showed a unique absorption peak using the pANTXR1 specific primers that the upstream primer pANTXR1KO-F included eight nucleotides knocked out (shown in Table S1), while that of in PAM-Tang KO cells had none. (B) The melting-curve in both of PAM-Tang wt cells and PAM-Tang KO cells showed a unique absorption peak using the β-actin specific primers.

**Oligonuclcotide list for primers**


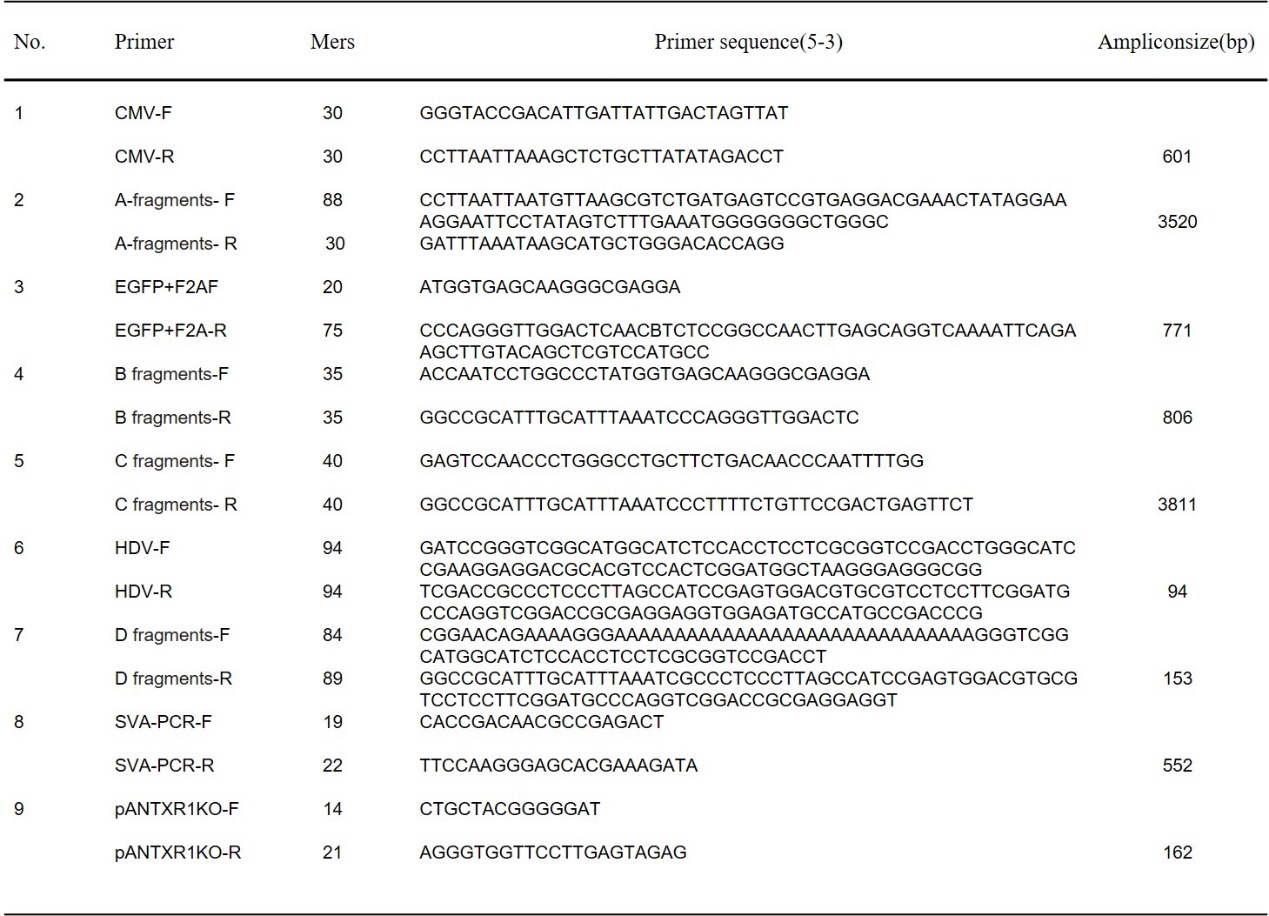


**Table S1. Oligonuclcotide list for primers used in cloning and the pANTXR1 detection specific primers for RT-qPCR method**
